# Associations Between Anxiety Symptoms and Health-Related Quality of Life: A Population-Based Twin Study in Sri Lanka

**DOI:** 10.1007/s10519-021-10051-1

**Published:** 2021-02-18

**Authors:** Zeynep Nas, Helena M. S. Zavos, Athula Sumathipala, Kaushalya Jayaweera, Sisira Siribaddana, Matthew Hotopf, Frühling V. Rijsdijk

**Affiliations:** 1 Social, Genetic & Developmental Psychiatry Centre, Institute of Psychiatry, Psychology and Neuroscience, King’s College London, London, UK; 2 Department of Psychology, Institute of Psychiatry, Psychology and Neuroscience, King’s College London, London, UK; 3 Institute for Research and Development, Colombo, Sri Lanka; 4 Faculty of Medicine & Allied Sciences, Rajarata University of Sri Lanka, Anuradhapura, Sri Lanka; 5 Psychological Medicine Department, Institute of Psychiatry, Psychology, and Neuroscience, King’s College London, London, UK; 6 NIHR Biomedical Research Centre for Mental Health at the South London and Maudsley NHS Foundation Trust, King’s College London, London, UK; 7 Research Institute for Primary Care and Health Sciences, Faculty of Health, Keele University, Keele, UK

**Keywords:** Anxiety, Quality of life, Twin study, Sex differences, Non-western samples

## Abstract

Anxiety not only concerns mental wellbeing but also negatively impacts other areas of health. Yet, there is limited research on (a) the genetic and environmental aetiology of such relationships; (b) sex differences in aetiology and (c) non-European samples. In this study, we investigated the genetic and environmental variation and covariation of anxiety symptoms and eight components of health-related quality of life (QoL), as measured by the short form health survey (SF-36), using genetic twin model fitting analysis. Data was drawn from the Colombo Twin and Singleton Study (COTASS), a population-based sample in Sri Lanka with data on twins (N = 2921) and singletons (N = 1027). Individual differences in anxiety and QoL traits showed more shared environmental (family) effects in women. Men did not show familial effects. Anxiety negatively correlated with all eight components of QoL, mostly driven by overlapping unique (individual-specific) environmental effects in both sexes and overlapping shared environmental effects in women. This is the first study in a South Asian population supporting the association between poor mental health and reduced QoL, highlighting the value of integrated healthcare services. Associations were largely environmental, on both individual and family levels, which could be informative for therapy and intervention.

**Supplementary Information:**

The online version contains supplementary material available at 10.1007/s10519-021-10051-1.

## Introduction

Anxiety symptoms are highly prevalent in the population (Mehta et al. 2003; Mallorquí-Bagué et al. 2016), and can be viewed as a continuum with healthy individuals on one end and those with anxiety disorders on the other. Anxiety symptoms impact not only emotional wellbeing, but are also associated with chronic health problems (Davies and Allgulander 2013; El-Gabalawy et al. 2014; Tang et al. 2017), bodily pain (Lerman et al. 2015), fatigue (Vassend et al. 2018) and sedentary lifestyles (Bélair et al. 2018; Stubbs et al. 2017; Vancampfort et al. 2018). Social skills and engagement in group activities are also reduced especially with a socially anxious profile (Scharfstein et al. 2011). Together, these limitations substantially impair quality of life (QoL). Yet, these overlaps are often undetected in healthcare settings, with physical health often taking priority. Understanding how anxiety symptoms are related to QoL carries importance for informing healthcare planning and prioritising both mental and physical wellbeing.

Anxiety symptoms, as measured by the GAD-7 (Generalised anxiety), STPI (State anxiety) and APQ (Anxious personality questionnaire), are heritable, with 20–70% of individual differences in symptoms attributable to genetic differences between people in the population (López‐Solà et al. 2014; Petkus et al. 2016; Malanchini et al. 2017). Importantly, heritability of anxiety and the relative influence of the environment shows developmental changes, such as genetic innovation arising later on in life (Lee et al. 2016; Petkus et al. 2016). Health-related QoL measures including physical activity (Carlsson et al. 2013; den Hoed et al. 2013), social functioning (McGue and Christensen 2007), fatigue and pain (Vassend et al. 2018) have also been the focus of twin studies. There is, however, limited twin research combining these different areas of health related QoL. Tapping into eight general domains, the short-form health survey (SF-36) is a valid and reliable assessment of health-related QoL. Yet, behaviour genetic research on this measure is sparse. An early twin study on male twins from the US suggests that 17–33% of the variance in the eight domains of the survey can be explained by genetic factors (Romeis et al. 2005). Environment shared by twins had small to negligible effects whereas unique environmental influences explained a large proportion of variance. A later study on Danish twins used a shortened version of the survey, yielding similar heritability estimates ranging 11–35% with most of the variance accounted for by unique environmental effects (Steenstrup et al. 2013). Yet, to our knowledge, no twin studies have combined anxiety with these QoL domains in a genetically informative sample.

There is little information on whether anxiety and health related QoL are correlated due to overlapping genetic or environmental factors i.e. share the same aetiological origin. It is also unknown whether there are sex differences in the aetiological overlap. Previous studies indicate females as disproportionately affected by anxiety symptoms with higher heritability estimates compared to males (Ask et al. 2014). Findings, however, are inconclusive. Studies report small to negligible sex differences in anxiety prevalence and its variance decomposition, again dependent on developmental time points (Lamb et al. 2010; Franić et al. 2010; Durbeej et al. 2019).

Another major limitation of previous work is that most of the evidence comes from western samples, and findings may not necessarily extrapolate across cultures. Twin studies conducted in non-western populations reveal differences in genetic and environmental influences. One such study conducted on Chinese twins (N = 712) finds a modest heritability (23%) for anxiety symptoms in late childhood, decreasing to a negligible effect at mid-adolescence (Zheng et al. 2016). Contrary to twin studies in the western world, shared environmental influences were found to increase substantially overtime. Another study conducted on 620 Chinese adolescent twin pairs yields a much lower estimate for the heritability of anxiety symptoms (9.9%) (Unger et al. 2011). No sex differences were observed in the two studies described. Yet, another study in Chinese children and adolescents (N = 1400) reports not only higher heritability (ranging 50% for self-report and 63% for parent reported anxiety), but also sex differences, whereby heritability of anxiety was higher in girls for self‐reported data, but higher in boys for parent‐reported data (Chen et al. 2015a). Modest-high heritability estimates (26–48%) were also obtained for studies using large Korean twin samples (Sung et al. 2011; Song et al. 2017, 2019). Despite these studies, research is still behind on the inclusion of South-Asian participants, especially at older age ranges.

The present study uses a South Asian population-based adult twin and singleton sample to investigate (i) the genetic and environmental variance components of anxiety symptoms and health related QoL; (ii) their phenotypic relationships; (iii) the extent to which overlapping genetic and environmental factors underlie their associations and (iv) sex differences in these parameters.

## Methods

### Sample

We used a representative population sample from the Colombo Twin and Singleton Study (COTASS), as part of the Sri Lankan twin registry (Sumathipala et al. 2013). This is a two-wave cohort study of twins and singletons residing in the Colombo district, a mix of urban and rural environments and home to ~ 2.3 million people. Both mental and physical health was assessed, with the first wave completed in 2005–2007 (Siribaddana et al. 2008) and the second phase (COTASS-2), acquired between 2012 and 2015 (Jayaweera et al. 2018). For this study, we used data from COTASS-2, with twins (N = 2921) and singletons (N = 1027) followed up from the original COTASS study through invitation letters and by telephone. Data collection was conducted during home visits. The sample consisted of a total of 3948 individuals (1676 males; 42.5% and 2272 females; 57.5%) (Table 1). Singletons were significantly older than the twin sample with a mean age of 51.46 and 39.88, respectively (t (1645.2) = − 22.17, p < 0.001).

**Table 1:** Number of individuals included in the analyses, by sex and zygosity group

Zygosity	Males	Females	Total number of individuals
MZ			
Number of individual twins in full pairs^a^	478	668	1263
Number of single twins	55	62	
DZ			
Number of individual twins in full pairs^a^	302	410	850
Number of single twins	63	75	
DZOS			
Number of individual twins in full pairs^a^	343	343	808
Number of single twins	47	75	
Singletons	388	639	1027
Total	1676	2272	3948

*MZ* monozygotic twins, *DZ* dizygotic twins, *DZOS* dizygotic opposite sex twins

^a^These are individuals who are part of a complete twin pair

All participants provided written informed consent. Individuals that did not understand the consent process or the questionnaires due to language barriers were excluded. Participants that had successfully completed one or more study parts were offered 750 LKR (approximately £3.50 GBP) to compensate for their time. COTASS received ethical approval from the Psychiatry, Nursing & Midwifery Research Ethics subcommittee, King’s College London, UK (reference number: PNM/10/11-124) and the ethical review committee at the Faculty of Medical Sciences, University of Sri Jayewardenepura, Sri Lanka (reference number: 596/11).

### Measures

#### Anxiety

Anxiety was measured using the *GAD-7* (Spitzer et al. 2006), which captures the presence of generalised anxiety, as indicated in the DSM, over the past 2 weeks. Participants indicated for 7 items how often they were bothered by problems such as ‘feeling nervous, anxious or on edge’ and ‘trouble relaxing’, ranging from 0 (Not at all) to 3 (nearly every day). A total anxiety score was derived, ranging from 0 to 21. Raw scores of 0–4 indicate minimal anxiety, 5–9 mild, 10–14 moderate and 15–21 indicating severe anxiety (Spitzer et al. 2006). The GAD-7 has been shown to have excellent psychometric properties, capturing anxiety symptoms in a reliable and valid way (Spitzer et al. 2006; Löwe et al. 2008; Hinz et al. 2017). We also yielded good internal consistency for the GAD-7 measure (Cronbach’s α = 0.87).

#### Health-related quality of life

The *Short Form Health Survey (SF-36)* was used to gauge health related quality of life (Ware and Sherbourne 1992). The 36-item scale measures eight domains of health: general health perceptions (five items); limitations of physical activities due to health problems (physical functioning; 10 items); limitations in usual activities due to physical health problems (role physical; four items); bodily pain (two items); vitality (energy/fatigue; four items); limitations in social activities due to health problems (social functioning; two items); mental health (emotional well-being; five items) and limitations in usual activities due to emotional problems (role emotional; three items). A final item, named self-reported health transition, is answered by the participant but is not included in the scoring process. Some items on the questionnaire are recoded so that the scores range from 0 to 100, with 100 representing the best state of health, and 0 indicating worst. After recoding, an average score is obtained from the number of items per domain. The SF-36 survey has been widely used, with demonstration of good reliability and validity (Mchorney et al. 1993). A good internal consistency was found, as averaged across the eight domains (Cronbach’s α = 0.82).

### Analyses

The classical twin design rests on the known genetic difference across monozygotic (MZ; identical) and dizygotic (DZ; non-identical) twins. MZ twins share 100% of their genes, whereas DZ twins share, on average, 50% of their segregating genes. MZ and DZ twins are assumed to have similar shared environments (e.g. in-utero experiences and parental upbringing) and so differences in similarity are attributed to their genetic differences. This information is used in biometrical structural equation modelling (SEM) to disentangle the variance of a trait into three latent influences: additive genetic (A), common environmental (C) contributing to similarity within twin pairs, and unique environmental factors (E), contributing to differences within twin pairs (including measurement error). This model can be extended to bivariate analyses, which further decomposes the covariance between two traits into A, C and E contributions. These aetiological correlations (denoted rA, rC and rE) indicate how much the A, C and E factors underlying individual differences in one trait also affect the other (Rijsdijk and Sham 2002). These correlations and the standardized variance components are then used to determine the extent to which the phenotypic correlation (rPh) between anxiety symptoms and each of the QoL scales is due to correlated A, C and E factors (rPh-A, rPh-C and rPh-E, respectively).

Furthermore, twin models can test for sex differences in the aetiology of traits and the aetiological overlap between traits. Including same-sex and opposite-sex twin pairs allows testing for (a) qualitative sex differences—different genetic and environmental factors influencing variance and covariance of traits across sex and (b) quantitative sex differences—whereby the same genetic and environmental factors influence variance/covariance but differ in magnitude across male and female twin pairs. We began with a full bivariate sex limitation model testing for both qualitative sex differences (first for A then for C) and quantitative sex differences in the variance and covariance of anxiety and QoL variables, allowing all parameters to vary across sex (full heterogeneity model). We follow this by testing for quantitative sex differences only. A non-significant decline in fit between the model allowing quantitative sex differences only indicates that there are no qualitative sex differences. This is followed by a nested homogeneity model which equates all A, C and E path estimates across males and females. To detect the best-fitting model, differences in minus twice log likelihood (-2LL) (distributed as χ^2^) were examined between nested models, in addition to the Akaike’s Information Criterion (AIC) whereby a lower AIC generally indicates a better fit (Rijsdijk and Sham 2002; Neale and Cardon 2013). We used scores on the anxiety and QoL scales as continuous variables, regressed by age and sex and log transformed to minimise skew. The only exception to this was social functioning, where a threshold liability model was fitted to a dichotomous variable in a combined ordinal-continuous analysis with anxiety symptoms. Twin model fitting was conducted using the OpenMx statistical package in R (Neale et al. 2016).

Singletons were also included in analyses. Although they cannot contribute to information on the A, C and E variance and covariance decomposition of the genetic model, they add information on the phenotypic variances and covariances of variables and are therefore included in the genetic analyses, just like incomplete singleton twins.

Prior to fitting genetic models, we ran a fully saturated model for each variable followed by a sub model in which variances were tested for equality across sex (Supplementary Table V, Sub 1 models). For seven of the nine variables, this constraint resulted in a significant reduction in fit. In the univariate genetic analyses, we therefore proceeded to testing scalar sex-limitation models, whereby the same aetiology is specified across sex but allowing differences in variances. For most variables, however, this scalar model was a poor fit in comparison to the quantitative heterogeneity model, except for physical functioning, emotional wellbeing, and pain. In the bivariate genetic analyses, we therefore fitted a hybrid model for anxiety and these scales, specifying male and female ACE components for anxiety and a scalar variance inequality ACE model for the scale variables. In addition, for the bivariate analysis of anxiety and the energy/fatigue scale, we used a hybrid model specifying a homogeneity model for this scale, as the univariate analyses indicated that a homogeneity model does not result in a significant reduction in fit. Bivariate model fit statistics are detailed in Supplementary Table VI.

We also conducted post-hoc MZ twin differences analyses (Pike et al. 1996), to investigate the unique environmental component further. As MZ twins do not differ in their genetic makeup and shared environment, any differences observed is an index of their unique environmental experiences. The MZ-difference design focuses on relative difference scores within twin pairs (twin 1 -twin 2). Here, we calculated difference scores on a number of stressful life events, as measured via the 56-item life-threatening experiences questionnaire culturally adapted for the Sri-Lankan population (Brugha and Cragg 1990). These difference scores are then correlated with relative difference scores on outcome measures (here being GAD-7 anxiety symptoms and the QOL scales). If MZ twins who experienced more stressful life events also showed higher levels of anxiety and lower levels of QOL than the co-twin who experienced less stressful life events, then we can infer that stressful life events might be components of unique environmental variances and covariances of anxiety and QOL.

## Results

### Descriptive statistics

Descriptive statistics on age, anxiety symptoms and QoL symptoms for each study group is detailed in Table 2. The majority of individuals had minimal or no anxiety, with 8.5% mild, 2.3% moderate and 1.6% with severe anxiety symptoms according to cut-offs provided by Spitzer et al (2006). The distribution of anxiety scores is given in Supplementary Fig. 1. Females reported significantly higher anxiety symptoms than males, with a mean score of 1.54 and 2.10 respectively (t (3852.3) = − 5.4, p < 0.001). Females also report lower health related QoL in comparison to males for all eight scales of the SF-36. Details of these formal tests can be found in Supplementary Table I**.**

**Table 2:** Means (SD) of Age, Anxiety symptoms & health related quality of life (QoL) measures

	MZM	DZM	MZF	DZF	DZOS	Singleton males	Singleton females
Age	37.53 (12.49)	39.41 (13.02)	39.21 (12.83)	43.09 (14.07)	40.28 (13.19)	52.48 (15.45)	50.84 (14.32)
Anxiety	1.53 (2.75)	1.29 (2.62)	1.90 (3.06)	2.01 (3.78)	1.83 (3.30)	1.85 (3.48)	2.36 (3.75)
General health	63.26 (14.97)	62.83 (14.39)	61.45 (16.35)	60.28 (16.94)	61.01 (15.88)	60.94 (15.04)	57.14 (17.71)
Physical functioning	93.82 (14.90)	93.95 (13.36)	88.53 (19.25)	88.05 (19.24)	91.39 (17.06)	87.12 (22.62)	82.94 (21.53)
Role of physical problems	86.11 (31.72)	88.67 (29.47)	81.97 (35.3)	79.24 (37.01)	82.21 (35.43)	79.86 (38.02)	78.21 (39.24)
Emotional wellbeing	78.85 (15.70)	80.5 (14.00)	77.36 (15.58)	76.83 (16.78)	78.57 (15.94)	80.44 (14.15)	76.25 (17.42)
Role of emotional problems	88.7 (28.88)	91.53 (25.10)	85.24 (31.9)	85.91 (31.89)	87.39 (30.65)	88.17 (30.31)	84.56 (34.44)
Energy/fatigue	74.3 (17.15)	75.3 (14.97)	74.56 (16.49)	73.80 (17.07)	74.21 (16.83)	71.16 (16.83)	69.63 (17.68)
Pain	88.7 (19.57)	90.3 (16.99)	85.46 (20.78)	83.56 (21.29)	86.34 (19.52)	87.64 (20.46)	86.50 (21.10)
Social functioning	89.93 (17.81)	91.31 (17.76)	88.5 (19.05)	88.31 (20.86)	89.65 (18.56)	89.22 (21.47)	89.00 (20.48)

The range of the anxiety scale = 0–21; The range of the total SF-36 sub-scales = 1–100

*MZM* monozygotic male twins, *MZF* monozygotic female twins, *DZM* dizygotic male twins, *DZF* dizygotic female twins, *DZOS* dizygotic opposite sex twins

Singletons had significantly higher self-reported anxiety symptoms compared to twins, with a mean score of 1.76 and 2.16 respectively (t (1604) =  − 3.18, p = 0.001). This effect remained even after accounting for age (t (1604.2) = − 3.55, p < 0.001). Twins and singletons also showed significant differences on four out of the eight health related QoL scales; general health (t (1712.9) = 5.09, p < 0.001), physical functioning (t (1503.8) = 8.36, p < 0.001), role limitations due to physical health problems (t (1632.4) = 3.17, p = 0.002) and energy/fatigue (t (1737.5) = 6.70, p < 0.001). The latter scale showed significant differences even after accounting for age (t (1733.3) = 4.29, p < 0.001).

### Phenotypic model fitting

We conducted phenotypic analyses for each variable. The cross-twin within-trait correlations (Table 3) suggested little heritability of anxiety across sex, with the MZ:DZ ratio roughly 1:1. The correlations also indicate the influence of shared environmental effects especially for females, since their correlations are significant. As with anxiety symptoms, the phenotypic correlations suggest little or no heritability for the QoL scales with MZ:DZ ratios roughly being 1:1, indicating the effects of shared environment as the familial factor contributing to the variance (individual differences) of the traits. Cross-twin cross-trait correlations can be found in Supplementary Table II and indicate (by the largely non-significant cross-twin cross-trait correlations), for males, unique-environmental sources of covariance between anxiety and QoL measures. For females, however, these correlations are all significant and roughly equal across MZ and DZ pairs, suggesting a shared-environmental (e.g. family environment) source of covariance between anxiety and QoL. The within-individual cross-trait correlations (rPh) were significant and negative between anxiety and all eight components of the health survey, ranging from -0.17 for physical functioning to -0.58 for emotional wellbeing (Supplementary Table III). The actual aetiological components of these results are estimated in the univariate and bivariate genetic models (below).

**Table 3:** Twin correlations (cross-twin within trait) (with 95% CIs)

QoL variable	MZM	DZM	MZF	DZF	DZOS
Anxiety	.09 (− .07/.25)	.08 (− .12/.26)	**.23** **(.12**/**.33)**	**.30** **(.17**/**.40)**	.01 (− .08/.11)
General health	**.25** **(.12**/**.37)**	**.27** **(.10**/**.41)**	**.31** **(.21**/**.40)**	**.34** **(.22**/**.45)**	.11 (.00/.22)
Physical functioning	.12 (− .13/.32)	**.47** **(.23**/**.61)**	**.38** **(.28**/**.47)**	**.26** **(.12**/**.38)**	**.22** **(.10**/**.33)**
Role of physical problems	.05 (− .11/.20)	.20 (− .01/.37)	**.26** **(.14**/**.36)**	**.30** **(.17**/**.41)**	.02 (− .08/.12)
Emotional wellbeing	**.23** **(.12**/**.34)**	.18 (− .01/.35)	**.31** **(.20**/**.41)**	**.23** **(.10**/**.34)**	.10 (.00/.21)
Role of emotional problems	**.19** **(.05**/**.31)**	.09 (− .14/.30)	**.19** **(.09**/**.30)**	**.32** **(.19**/**.44)**	.05 (− .05/.16)
Energy/fatigue	**.18** **(.05**/**.30)**	**.20** **(.01**/**.37)**	**.27** **(.16**/**.37)**	**.28** **(.15**/**.40)**	**.19** **(.09**/**.29)**
Pain	.11 (− .02/.24)	**.36** **(.19**/**.50)**	**.17** **(.06**/**.27)**	**.24** **(.12**/**.35)**	.05 (− .07/.16)
Social functioning	**.42** **(.22**/**.60)**	**.43** **(.16**/**.65)**	**.43** **(.27**/**.57)**	**.49** **(.28**/**.66)**	**.32** **(.15**/**.47)**

Significant twin correlations are given in bold (as indicated by 95% CI not crossing zero). Please note that cross-twin cross trait correlations can be found in Supplementary Table 2

*MZM* monozygotic male twins, *MZF* monozygotic female twins, *DZM* dizygotic male twins, *DZF* dizygotic female twins, *DZOS* dizygotic opposite sex twins

### Univariate model fitting

Table 4 details the standardized variance components of all variables. For anxiety symptoms, there was little indication of heritability across sex. In females, a significant proportion of variance in anxiety was explained by shared environment (25%). Unique environmental influences explained a large proportion of variance in anxiety symptoms for males (91%) and females (75%). This was also the case for the SF-36 scales, with a large proportion of variance explained by unique environmental influences across sex (68–93%). We found significant genetic influence (heritability) for emotional wellbeing (23%), which fit a scalar model so equated across sex. General health, role of emotional problems and social functioning all showed significant amount of shared environmental influences in females (23–28%). The energy/fatigue scale also showed a significant influence of shared environment (22%), which was equal across sex due to a homogeneity model fitting best.

**Table 4:** Standardised variance components of Anxiety symptoms and health related quality of life (QoL) measures in males and females (with 95% CIs) obtained from univariate analyses

QoL variable	Sex	Aetiology (95% CI)		
		**A (h**^**2**^**)**	**C (c**^**2**^**)**	**E (e**^**2**^**)**
Anxiety	M	.09 (.00/.24)	.00 (.00/.09)	**.91** **(.76**/**1)**
	F	.00 (.00/.21)	**.25** **(.06**/**.33)**	**.75** **(.66**/**.83)**
General health	M	.17 (.00/.36)	.10 (.00/.29)	**.73** **(.62**/**.85)**
	F	.04 (.00/.30)	**.28** **(.05**/**.39)**	**.68** **(.59**/**.76)**
Physical functioning (scalar model)	M | F	.16 (.00/.39)	.16 (.00/.33)	**.68** **(.60**/**.77)**
Role of physical problems	M	.09 (.00/.23)	.00 (.00/.15)	**.91** **(.77**/**1)**
	F	.00 (.00/.31)	.27 (.00/.35)	**.73** **(.64**/**.82)**
Emotional wellbeing (scalar model)	M | F	**.23** **(.02**/**.34)**	.04 (.00/.21)	**.73** **(.66**/**.81)**
Role of emotional problems	M	.17 (.00/.30)	.02 (.00/.16)	**.81** **(.69**/**.95)**
	F	.00 (.00/.16)	**.23** **(.08**/**.31)**	**.77** **(.69**/**.85)**
Energy/fatigue (scalar model)	M | F	.01 (.00/.26)	**.22** **(.05**/**.28)**	**.77** **(.70**/**.83)**
Pain (scalar model)	M | F	.00 (.00/.19)	.15 (.00/.21)	**.85** **(.77**/**.90)**
Social functioning	M	.07 (.00/.23)	.00 (.00/.00)	**.93** **(.76**/**.99)**
	F	.02 (.00/.18)	**.23** **(.09**/**.33)**	**.75** **(.67**/**.84)**

Significant parameters are given in bold (as indicated by 95% CI not crossing zero). These estimates are obtained from the univariate heterogeneity sex limitation analysis. Note that for three variables (physical functioning, emotional wellbeing, and pain) we fit models which specify same aetiology across sex, but allowing different variances. Also note that for the energy/fatigue variable, the homogeneity model fit best, meaning that the aetiology was equal across sex

*M* males, *F* females, *A* additive genetic influences, *C* common environmental influences, *E* unique environmental influences

### Bivariate genetic model fitting

Full sex limitation models were fit to the data, specifying both qualitative and quantitative sex differences (first for A then for C; heterogeneity models), which allows parameters to be estimated separately across males and females. Supplementary Table VI details these model fit comparisons. Overall, there were no significant differences between the models specifying quantitative sex differences only and models allowing for qualitative sex differences. The exception to this was the analyses between anxiety-pain, in which there was some evidence for qualitative sex differences in C, though with less reliable standard errors. The C correlations obtained from this qualitative C model were non-significant, detailed in Supplementary Table VII. Homogeneity sub-models, whereby path estimates for A, C and E are equated across males and females, were therefore compared to the quantitative heterogeneity models to examine whether the magnitudes of A, C and E effects on anxiety and QoL components differ across sex. A significant decline in fit indicates sex differences. All eight bivariate genetic homogeneity models resulted in a highly significant reduction in fit. This suggests that the magnitude of genetic and environmental factors influencing anxiety and health related QoL measures were quantitatively different across sex.

### Decomposing covariances

Sex differences are evident in the phenotypic correlation breakdown (Table 5). The table decomposes the negative phenotypic correlations into parts due to correlated A (rPh-A), C (rPh-C) and E (rPh-E) factors. In females, there was a large contribution of shared and unique environmental effects, whereas in males the main contributor was unique environmental influences.

**Table 5:** Phenotypic correlations between anxiety symptoms and health related quality of life (QoL) measures with their corresponding A, C and E components (with 95% CIs) in males and females

QoL variable	Sex	Phenotypic correlation (rPh) (95% CI)	rPh components (95% CI)		
			rPh-A	rPh-C	rPh-E
General health	M	− **.29** **(**− **.33**/− **.24)**	− .01 (− .11/.09)	.00 (− .05/.03)	− **.28** **(**− **.38**/− **.18)**
	F	− **.26** **(**− **.30**/− **.22)**	− .02 (− .10/.08)	− **.12** **(**− **.22**/− **.04)**	− **.12** **(**− **.18**/− **.06)**
Physical functioning	M	− **.22** **(**− **.27**/− **.17)**	− .11 (− .22/.05)	.00 (− .13/.04)	− **.11** **(**− **.21**/− **.01)**
	F	− **.17** **(**− **.21**/− **.13)**	− .02 (− .18/.10)	− .08 (− .16/.06)	− **.07** **(**− **.14**/− **.01)**
Role of physical problems	M	− **.27** **(**− **.31**/− **.22)**	− .05 (− .15/.05)	.00 (− .03/.04)	− **.22** **(**− **.33**/**.11)**
	F	− **.26** **(**− **.30**/− **.22)**	.00 (− .08/.10)	− **.13** **(**− **.23**/− **.05)**	− **.13** **(**− **.19**/− **.07)**
Emotional wellbeing	M	− **.52** **(**− **.56**/− **.49)**	− .05 (− .13/.01)	− .04 (− .11/.01)	− **.43** **(**− **.50**/− **.35)**
	F	− **.58** **(**− **.60**/− **.55)**	− .07 (− .14/.00)	− **.14** **(**− **.20**/− **.06)**	− **.37** **(**− **.43**/− **.31)**
Role of emotional problems	M	− **.43** **(**− **.47**/− **.39)**	− .10 (− .20/.02)	.00 (− .08/.02)	− **.33** **(**− **.45**/− **.22)**
	F	− **.46** **(**− **.49**/− **.42)**	.00 (− .12/.04)	− **.22** **(**− **.29**/− **.11)**	− **.24** **(**− **.30**/− **.18)**
Energy/fatigue	M	− **.40** **(**− **.44**/− **.37)**	− .08 (− .20/.03)	− .03 (− .14/.04)	− **.29** **(**− **.36**/− **.23)**
	F	− **.44** **(**− **.47**/− **.40)**	.02 (− .16/.06)	− **.19** **(**− **.24**/− **.03)**	− **.27** **(**− **.33**/− **.21)**
Pain	M	− **.31** **(**− **.35**/− **.26)**	− .03 (− .13/.06)	− .04 (− .11/.02)	− **.24** **(**− **.33**/− **.16)**
	F	− **.29** **(**− **.33**/− **.25)**	.01 (− .15/.07)	− .13 (− .19/.00)	− .15 (− .23/− .08)
Social functioning	M	− **.43** **(**− **.48**/− **.38)**	− .15 (− .26/.07)	− .01 (− .25/.02)	− **.27** **(**− **.39**/− **.16)**
	F	− **.44** **(**− **.49**/− **.40)**	.06 (− .33/.06)	− .18 (− .30/.06)	− **.20** **(**− **.29**/− **.12)**

Significant parameters given in bold, as indicated by 95% CI not crossing zero

*M* males, *F* females, *rPh* phenotypic correlation, *rPh-A* additive genetic component of rPh, *rPh-C* common environmental component of rPh, *rPh-E* Unique environmental component of rPh

We found significant shared environmental correlations (rC) in females in four out of the eight analyses, indicating that common environmental factors (e.g. family environment) that contributed to higher scores on anxiety also contributed to lower scores on the QoL variables. Unique environmental correlations (rE) were all significant and negative in males and females. Our bivariate analyses did not yield any significant genetic correlations (rA) between anxiety and any of the QoL components. Estimates of aetiological correlations of the bivariate models (rA, rC and rE) can be found in Supplementary Table IV. Full bivariate model fit statistics can be found in Supplementary Table VI.

## Discussion

This is the first twin study examining associations between anxiety symptoms and health related quality of life (QoL) in a South-Asian population. Our study adds to the limited literature surrounding their genetic and environmental aetiology, aetiological correlations, and sex differences. Females reported higher levels of anxiety symptoms and lower self-reported QoL, consistent with research in western populations (Ask et al. 2014; Garratt and Stavem 2017).

### Aetiology

Unique (individual-specific) environmental influences explained the majority of variance in anxiety symptoms in males and females and across the health related QoL measures (68–93%). The large contribution of unique environmental effects (including measurement error) is in line with previous work conducted in western samples (Romeis et al. 2005; Steenstrup et al. 2013). There was no significant heritability for anxiety in males or females, making it a stark contrast to estimates coming from western populations (Trzaskowski et al. 2012; López‐Solà et al. 2014; Malanchini et al. 2017). This is also in contrast with data from other Asian samples, including Chinese (Chen et al. 2015a) and Korean samples (Sung et al. 2011). This study, however, is conducted in a South Asian sample, and results should be interpreted in this socio-cultural context.

Overall, genetic factors explained 0–23% of variance in health related QoL. Out of the QoL measures, only emotional wellbeing showed significant heritability. This is comparable to a previous study on male twins from the US (Romeis et al. 2005), and point estimates are similar to a Danish twin sample although using a shortened version of the health survey (Steenstrup et al. 2013). More genetically informative research is required, both in western and non-western samples, to confirm these findings.

The most prominent sex difference is the significant shared environmental influences on females’ anxiety symptoms, consistent with data coming from Chinese samples (Chen et al. 2015a, 2016). Several QoL measures in females also show significant shared environmental influence, implying female-specific, common socio-cultural factors underlying individual differences in these traits. The low heritability estimates are remarkable and could reflect a more variable environment in Asian cultures impacting on mental well-being, particularly for men. Collectivist social norms may also contribute to attenuated heritability estimates, as observed with other phenotypes (Chen et al. 2015b). Environmental trauma is also worth noting, seeing as Sri Lanka was affected by a prolonged civil war (Ball et al. 2009) and a Tsunami in 2004. Our findings therefore reinforce the notion that genetic and environmental influence can be attenuated or amplified across cultural context and environmental variability.

### Phenotypic and aetiological correlations

Anxiety was significantly negatively correlated with all eight components of health related QoL. Correlation estimates were similar across sex, and the most important factor explaining these correlations are environmental effects unique to an individual (including measurement error). However, apart from this source of covariance, in females, we find evidence for influence of overlapping shared (family) environmental effects. We did not find evidence for overlapping genetic factors in these phenotypic correlations.

We found significant shared-environmental correlations in females, with most being negative, i.e. indicating family environmental influences that may increase anxiety symptoms and decrease health related QoL. Genetic correlations between anxiety and QoL measures were not significant, indicating that there is not likely to be a common genetic liability or genetic pleiotropic effects. All unique environmental correlations were significant and negative. Hence, the same environment exclusive to an individual can increase anxiety, and also decrease perceived QoL. As the role of the unique environment was so substantial, we decided to investigate this further, conducting post-hoc MZ twin differences analysis (Pike et al. 1996). Briefly, the method is used to isolate unique environmental influences by using relative difference scores for an environmental measure (e.g. stressful life events) and correlate this with difference scores on an outcome variable (e.g. anxiety). We found a positive correlation (r = 0.22, p < 0.001), indicating that those who experience more stressful life events also experience more anxiety, and as MZ twins are identical in terms of their genetics and shared environment, this association can be interpreted as truly environmental. To get an indication for the SF-36 scales, we ran the same analysis using the general health domain. We found a negative correlation (r = − 0.09, p = 0.04), indicating that those who experience more stressful life events also report a lower general health-related quality of life. Though it is worth noting that these correlations only explain a small amount of variance (< 5%). It may also be worth investigating other factors e.g. physical comorbidities that may explain the covariance between anxiety-physical health.

### Strengths

A major strength of our study is the use of a large, representative population-based twin registry based in Sri Lanka, especially considering the limited twin studies in non-western populations. In addition, we used a widely accepted quantitative measure of anxiety symptoms, which not only provides gain in statistical power (Gottschalk and Domschke 2017), but is in line with recent efforts to capture the dimensional nature of traits. As opposed to a categorical approach, symptoms may better conceptualize the anxiety spectrum, help identify those at risk especially individuals that do not meet criteria for a diagnosis (Keough et al. 2010). Our sample also included comparable singletons, further improving power, and was split by sex, revealing significant sex differences in both the aetiology and associations between anxiety and QoL.

### Limitations + future directions

We employed a cross-sectional design and are therefore limited in drawing inferences on causality. Higher anxiety may reduce QoL or a poor QoL may in turn increase anxiety symptoms. A longitudinal twin design and/or extensions to the twin model can better disentangle direction of effects and determine whether genetic and environmental influences increase or attenuate overtime (Kendler et al. 2008). Tracking developmental changes in anxiety and QoL can also be useful for designing interventions at the appropriate time. In addition, self-report questionnaires, though commonly used, gauge the presence of anxiety symptoms and perceived QoL rather than establish any diagnoses. Our study should therefore be extended to clinical populations and worth replicating in other South Asian samples to test whether these effects are generalisable. In addition, we did not yield qualitative sex differences, except for marginal (albeit less reliable) effects for C influences in the anxiety- pain analyses. Overall, the same genes and environments seem to be operating across men and women, only with different magnitudes of effect. We may, however, have been underpowered to detect qualitative sex differences, hence future work using larger sample sizes may provide sufficient statistical power to detect this. Furthermore, we find that twins differ from singletons on anxiety and several health related QoL scales even after accounting for age differences. This is also a characteristic of the main COTASS sample (Jayaweera et al. 2018) and might indicate that the twin modelling results may not necessarily extrapolate to the general Sri Lankan population. Additionally, we have attempted to characterise QoL through eight domains, which may not capture its full complexity. Findings must therefore be interpreted within the context of these specific domains rather than generalising to overall QoL. It is also worth noting that confidence intervals (particularly surrounding aetiological correlations) are wide. Replication in larger sample sizes can improve precision in estimates and in drawing more reliable conclusions.

In addition, although we did find a large contribution of unique environmental influences, this component also includes measurement error. As we ran bivariate models, correlated measurement error is also worth noting. Language barriers could normally influence this, although we overcame this by ensuring that participants had sufficient language proficiency to take part. As with other studies, however, response bias could affect questionnaire reporting and potentially inflate the non-shared environmental influence. Studies conducted in western populations using the SF-36 and SF-12 scales also find large influence of the unique environment (Romeis et al. 2005; Steenstrup et al. 2013), suggesting that this may be a limitation of the scale. Our findings should therefore be viewed in context of potential measurement error.

One of the most important future application for research of this kind is to inform healthcare planning, to form integrated healthcare systems for mental and physical health (Thornicroft et al. 2019). There are already various barriers to identifying and preventing anxiety in primary care, including stigma, masking/diagnostic overshadowing and prioritising physical diagnoses (Barnes et al. 2019).Screening for both mental, physical and QoL domains in primary care offers a holistic approach, recognising and preventing health issues as early as possible (Firth et al. 2019).

In conclusion, severity of anxiety symptoms was significantly associated with poorer health related quality of life. We find significant sex differences in both the variance and covariance of these traits. For women, individual differences in anxiety and QoL measures were explained largely by shared and unique environmental factors whereas men mostly show evidence of unique environmental influence. In terms of covariances between anxiety and QoL, we find significant overlapping common environmental correlations in females, suggesting the importance of the environment shared, e.g. within families. The unique environment experienced by an individual (including measurement error) had a large contribution to trait variances as well as covariances across sex. Our study is a first considering a behaviour genetic approach combining anxiety and QoL in a south Asian context. Findings have implications for cross-cultural behaviour genetic research and indicate the importance of therapeutic interventions focusing on the wider environment of an individual.

## Supplementary Information

Below is the link to the electronic supplementary material.

Supplementary information 1 (DOCX 124 kb)
