# Supplementary material for: Associations Between Anxiety Symptoms and Health-Related Quality of Life: A Population-Based Twin Study in Sri Lanka
Source: Behav Genet. 2021 Feb 18;51(4):394–404. doi: 10.1007/s10519-021-10051-1 (PMC8225527; doi:10.1007/s10519-021-10051-1)
Supplement: Supplementary file 1 — Supplementary information 1 (DOCX 124 kb) [file 10519_2021_10051_MOESM1_ESM.docx]

**Associations Between Anxiety Symptoms And Health-Related Quality Of Life: A Population-Based Twin Study In Sri Lanka**

***Supplementary material***

**Supplementary Figure 1.** Distribution of GAD-7 anxiety scores in the study sample

**
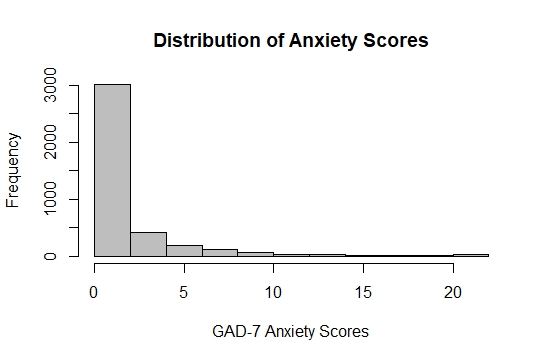
**

**Please note that the GAD-7 anxiety measure was log transformed prior to analyses to minimise skewness. Before transformation: Skewness = 3.02, Kurtosis = 10.87. After transformation: Skewness = .82, Kurtosis = .04.**

**Supplementary Table I. Welch’s t-tests for anxiety symptoms and SF-36 scales.**

| Variable | Mean  Males | Mean  Females | t | df | p | 95%CI |
| --- | --- | --- | --- | --- | --- | --- |
| GAD-7 | 1.54 | 2.10 | -5.40 | 3852.3 | **<.001** | -.76 , -0.36 |
| GENERAL HEALTH | 62.46 | 59.62 | 5.54 | 3757.9 | **<.001** | 1.83, 3.85 |
| PHYSICAL FUNCTIONING | 92.28 | 86.93 | 9.03 | 3822.1 | **<.001** | 4.18, 6.51 |
| ROLE OF PHYSICAL PROBLEMS | 85.13 | 79.67 | 4.82 | 3755.2 | **<.001** | 3.24, 7.68 |
| EMOTIONAL WELLBEING | 79.87 | 76.88 | 5.93 | 3772.6 | **<.001** | 2.00, 3.98 |
| ROLE OF EMOTIONAL PROBLEMS | 89.40 | 85.18 | 4.30 | 3797.2 | **<.001** | 2.30, 6.14 |
| ENERGY/FATIGUE | 73.99 | 72.81 | 2.17 | 3645.5 | **.03** | .11, 2.25 |
| PAIN | 88.73 | 85.11 | 5.63 | 3741.6 | **<.001** | 2.36, 4.88 |
| SOCIAL FUNCTIONING | 90.14 | 88.69 | 2.32 | 3642.8 | **.02** | .23, 2.68 |

**Supplementary Table II.** Cross-twin cross-trait correlations between Anxiety symptoms and health related QoL measures for each of the sex-by-zygosity twin groups (with 95% CIs)

| QoL VARIABLE | MZM | DZM | MZF | DZF | DZOS |
| --- | --- | --- | --- | --- | --- |
| GENERAL HEALTH | .00  (-.10 / .10) | -.06  (-.19 / .08) | **-.14**  **(-.21 / -.06)** | **-.13**  **(-.21 / -.04)** | .01  (-.07 / .09) |
| PHYSICAL FUNCTIONING | -.09  (-.22 /.05) | -.07  (-.21 / .08) | **-.09**  **(-.16 / -.01)** | **-.16**  **(-.24 / -.06)** | .01  (-.07 / .09) |
| ROLE OF PHYSICAL  PROBLEMS | -.05  (-.17 / .06) | -.08  (-.21 / .08) | **-.12**  **(-.19 / -.03)** | **-.15**  **(-.24 / -.06)** | -.01  (-.09 / .06) |
| EMOTIONAL WELLBEING | -.09  (-.20 / .02) | -.14  (-.28 / .01) | **-.24**  **(-.32 / -.15)** | **-.22**  **(-.31 / -.12)** | -.01  (-.09 / .08) |
| ROLE OF EMOTIONAL  PROBLEMS | -.09  (-.20 / .02) | -.10  (-.25 / .07) | **-.19**  **(-.27 / -.11)** | **-.26**  **(-.35 / -.17)** | -.06  (-.14 / .01) |
| ENERGY/FATIGUE | -.07  (-.18 / .04) | -.04  (-.18 / .12) | **-.22**  **(-.29 / -.13)** | **-.21**  **(-.30 / -.11)** | -.05  (-.13 / .02) |
| PAIN | -.06  (-.16 / .05) | **-.18**  **(-.30 / -.04)** | **-.13**  **(-.20 / -.05)** | **-.19**  **(-.27 / -.10)** | .00  (-.08 / .08) |
| SOCIAL  FUNCTIONING | -.12  (-.25 / .01) | **-.26**  **(-.41 / -.09)** | **-.22**  **(-.31 / -.12)** | **-.25**  **(-.36 / -.14)** | -.08  (-.17 / .02) |

*MZM = monozygotic male twins; MZF = monozygotic female twins; DZM = dizygotic male twins; DZF = dizygotic female twins; DZOS = dizygotic opposite sex twins. Significant correlations are given in bold (indicated by 95% CI not crossing zero).*

Please note that although the cross-twin within trait MZ/DZ twin correlations for general health in males (Table 2 main text) have a 1:1 ratio (indicating that trait aetiologies should largely be due to common environmental effects), we find a significant heritability for males. This is most likely due to the DZOS twins in the model (note that the DZOS twin correlation is small and non-significant), causing a shift in the mean estimates for the correlation in DZ males, leading to a larger difference in the MZ/DZ ratio and therefore a significant heritability estimate. In contrast, the cross-twin cross-trait MZ/DZ correlations (as seen here) for females are significant and have a ratio of 1:1, meaning that the familial effect explaining covariance between Anxiety and these traits is the shared (family) environment, which is what we also see in the results of the ACE model. The mostly non-significant cross-twin cross-trait correlations in males indicate ‘E’ to be the source of covariance between Anxiety and these traits.

**Supplementary Table III.** Phenotypic correlations between anxiety symptoms and health-related quality of life (QoL) variables in males and females (95% CIs)

| QoL VARIABLE | Sex | Phenotypic  Correlation (95%CI) |
| --- | --- | --- |
|  |  |  |
| GENERAL HEALTH | M | **-.29**  **(-.33 / -.25)** |
|  | F | **-.26**  **(-.30 / -.22)** |
| PHYSICAL  FUNCTIONING | M | **-.21**  **(-.26 / -.17)** |
|  | F | **-.17**  **(-.21 / -.13)** |
| ROLE OF PHYSICAL  PROBLEMS | M | **-.27**  **(-.31 / -.22)** |
|  | F | **-.26**  **(-.30 / -.22)** |
| EMOTIONAL  WELLBEING | M | **-.52**  **(-.56 / -.49)** |
|  | F | **-.58**  **(-.61 / -.55)** |
| ROLE OF EMOTIONAL  PROBLEMS | M | **-.43**  **(-.47 / -.39)** |
|  | F | **-.46**  **(-.49 / -.42)** |
| ENERGY/FATIGUE | M | **-.40**  **(-.44 / -.36)** |
|  | F | **-.44**  **(-.47 / -.41)** |
| PAIN | M | **-.31**  **(-.35 / -.26)** |
|  | F | **-.29**  **(-.33 / -.25)** |
| SOCIAL  FUNCTIONING | M | **-.43**  **(-.48 / -.38)** |
|  | F | **-.44**  **(-.49 / -.40)** |

*M = Males, F = Females. Significant correlations are given in bold (as indicated by 95% CI not crossing zero)*

| QoL VARIABLE | Sex | Correlations with anxiety (95% CI) | | |
| --- | --- | --- | --- | --- |
|  |  | **rA** | **rC** | **rE** |
| GENERAL  HEALTH | M | -.05  (-.99 / 1) | -.99  (-.99 /-.99) | **-.34**  **(-.45 / -.23)** |
|  | F | -1  (-1 / -1) | **-.45**  **(-1 / -.18)** | **-.17**  **(-.25 / -.09)** |
| PHYSICAL  FUNCTIONING | M | -.99  (-.99 / -.99) | .15  (-1 / .99) | **-.14**  **(-.26 / -.02)** |
|  | F | -.12  (-.99 / .74) | -1  (-1 / -1) | **-.11**  **(-.20 / -.01)** |
| ROLE OF PHYSICAL  PROBLEMS | M | -.63  (-1 / 1) | .99  (.99 / .99) | **-.24**  **(-.35 / -.13)** |
|  | F | .99  (.99 / .99) | **-.49**  **(-1/ -.26)** | **-.17**  **(-.25 / -.09)** |
| EMOTIONAL  WELLBEING | M | -.99  (-.99 / .99) | -1  (-1 / -1) | **-.50**  **(-.57 / -.43)** |
|  | F | -.99  (-.99 / -.99) | **-.99**  **(-.99 / -.57)** | **-.49**  **(-.55 / -.42)** |
| ROLE OF EMOTIONAL  PROBLEMS | M | -.84  (-1 / 1) | -.99  (-.99 / -.99) | **-.38**  **(-.48 / -.28)** |
|  | F | .99  (.99 / .99) | **-.88**  **(-.99 / -.69)** | **-.32**  **(-.39 / -.25)** |
| ENERGY/FATIGUE | M \| F | -1  (-1 / -1) | -1  (-1 / -1) | **-.35**  **(-.41 / -.29)** |
| PAIN | M | -.99  (-.99 / -.99) | -1  (-1 / -1) | **-.26**  **(-.36 / -.17)** |
|  | F | -.16  (-.99 / .99) | -.99  (-.99 / -.99) | **-.20**  **(-.28 / -.11)** |
| SOCIAL  FUNCTIONING | M | -1  (-1 / 1) | -1  (-1 / 1) | **-.38**  **(-.52 / -.24)** |
|  | F | -.60  (-1 / 1) | -.78  (-1 / 1) | **-.32**  **(-.44 / -.20)** |

**Supplementary Table IV.** A, C and E correlations between Anxiety symptoms and health related QoL measures (with 95% CIs)*M= Males; F= Females. rA = Genetic correlation; rC= Common environmental correlation; rE= Unique environmental correlation.* *Significant correlations are given in bold (as indicated by 95% CI not crossing zero). Note that these estimates are obtained from the best fitting bivariate ACE model .Also note that there is one set of aetiological correlations for the anxiety-energy/fatigue analysis as this was set to be a hybrid ACE model specifying a homogeneity model for the energy/fatigue scale.*

**Supplementary Table V**. Univariate model fit statistics

| Variable | Model | ep | -2LL | df | AIC | ΔLL | Δdf | p |
| --- | --- | --- | --- | --- | --- | --- | --- | --- |
| ANXIETY | Sat | 9 | 11892.76 | 3842 | 4208.76 | - | - | - |
|  | Sub1 | 8 | 12028.61 | 3843 | 4342.61 | 135.84 | 1 | 2.16E-31 |
|  | **HetACE** | **8** | **11893.64** | **3843** | **4207.64** | **-** | **-** | **-** |
|  | ScACE | 6 | 11906.77 | 3845 | 4216.77 | 13.13 | 2 | <.01 |
|  | HomACE | 5 | 12038.59 | 3846 | 4346.59 | 144.95 | 3 | 3.24E-31 |
| GENERAL HEALTH | Sat | 9 | 14416.67 | 3847 | 6722.67 | - | - | - |
|  | Sub1 | 8 | 14441.33 | 3848 | 6745.33 | 24.66 | 1 | 6.84E-07 |
|  | **HetACE** | **8** | **14418.50** | **3848** | **6722.50** | **-** | **-** | **-** |
|  | ScACE | 6 | 14425.37 | 3850 | 6725.37 | 6.86 | 2 | .03 |
|  | HomACE | 5 | 14448.76 | 3851 | 6746.76 | 30.25 | 3 | 1.22E-06 |
| PHYSICAL FUNCTIONING | Sat | 9 | 11809.19 | 3847 | 4115.19 | - | - | - |
|  | Sub1 | 8 | 11840.96 | 3848 | 4144.962 | 31.77 | 1 | 1.73E-08 |
|  | HetACE | 8 | 11814.39 | 3848 | 4118.39 | - | - | - |
|  | **ScACE** | **6** | **11818.52** | **3850** | **4118.52** | **4.12** | **2** | **.13** |
|  | HomACE | 5 | 11851.09 | 3851 | 4149.09 | 36.69 | 3 | 5.34E-08 |
| ROLE OF PHYSICAL  PROBLEMS | Sat | 9 | 20608.09 | 3847 | 12914.09 | - | - | - |
|  | Sub1 | 8 | 20626.69 | 3848 | 12930.69 | 18.60 | 1 | 1.61E-05 |
|  | **HetACE** | **8** | **20610.31** | **3848** | **12914.31** | **-** | **-** | **-** |
|  | ScACE | 6 | 20622.93 | 3850 | 12922.93 | 12.62 | 2 | <.01 |
|  | HomACE | 5 | 20639.55 | 3851 | 12937.55 | 29.24 | 3 | 1.99E-06 |
| EMOTIONAL WELLBEING | Sat | 9 | 14415.11 | 3847 | 6721.11 | - | - | - |
|  | Sub1 | 8 | 14432.29 | 3848 | 6736.29 | 17.18 | 1 | 3.41E-05 |
|  | HetACE | 8 | 14415.39 | 3848 | 6719.39 | - | - | - |
|  | **ScACE** | **6** | **14417.66** | **3850** | **6717.66** | **2.27** | **2** | **.32** |
|  | HomACE | 5 | 14434.05 | 3851 | 6732.05 | 18.65 | 3 | <.01 |
| ROLE OF EMOTIONAL PROBLEMS | Sat | 9 | 14263.04 | 3847 | 6569.04 | - | - | - |
|  | Sub1 | 8 | 14284.18 | 3848 | 6588.18 | 21.14 | 1 | 4.27E-06 |
|  | **HetACE** | **8** | **14265.77** | **3848** | **6569.77** | **-** | **-** | **-** |
|  | ScACE | 6 | 14272.17 | 3850 | 6572.17 | 6.40 | 2 | .04 |
|  | HomACE | 5 | 14292.23 | 3851 | 6590.23 | 26.46 | 3 | 7.63E-06 |
| ENERGY/  FATIGUE | Sat | 9 | 11752.72 | 3847 | 4058.72 | - | - | - |
|  | Sub1 | 8 | 11754.83 | 3848 | 4058.83 | 2.11 | 1 | 0.15 |
|  | HetACE | 8 | 11752.96 | 3848 | 4056.96 | - | - | - |
|  | **ScACE** | **6** | **11754.86** | **3850** | **4054.86** | **1.90** | **2** | **.39** |
|  | HomACE | 5 | 11756.50 | 3851 | 4054.50 | 3.54 | 3 | .32 |
| PAIN | Sat | 9 | 17986.93 | 3848 | 10290.93 | - | - | - |
|  | Sub1 | 8 | 18002.20 | 3849 | 10304.20 | 15.26 | 1 | 9.35E-05 |
|  | HetACE | 8 | 17994.79 | 3849 | 10296.79 | - | - | - |
|  | **ScACE** | **6** | **17997.04** | **3851** | **10295.04** | **2.25** | **2** | **.33** |
|  | HomACE | 5 | 18012.94 | 3852 | 10308.94 | 18.15 | 3 | <.01 |
| SOCIAL FUNCTIONING | Sat | 9 | 4185.07 | 3888 | -3590.93 | - | - | - |
|  | Sub1 | 8 | 4185.61 | 3889 | -3592.39 | 0.54 | 1 | .46 |
|  | **HetACE** | **9** | **4187.21** | **3890** | **-3592.79** | **-** | **-** | **-** |
|  | HomACE | 6 | 4375.23 | 3893 | -3410.77 | 188.02 | 3 | 1.63E-40 |

Note that the homogeneity model was compared to the heterogeneity ACE model. The scalar model (testing differences in variance across sex) was compared to the heterogeneity ACE model. No scalar model was fitted for the social functioning variable as we fitted a liability threshold model with unit variance for males and females. Best fitting models are indicated in bold.

**Supplementary table VI.** Bivariate model fit statistics

| Variable | Model | ep | -2LL | df | AIC | ΔLL | Δdf | p |
| --- | --- | --- | --- | --- | --- | --- | --- | --- |
| GENERAL HEALTH | Sat | 25 | 26014.53 | 7682 | 10650.53 | - | - | - |
|  | Qual A | 26 | 26017.43 | 7681 | 10655.43 | - | - | - |
|  | Qual C | 26 | 26015.26 | 7681 | 10653.26 | - | - | - |
|  | **HetACE** | **22** | **26017.45** | **7685** | **10647.45** | .02 | 4 | .99 |
|  |  |  |  |  |  | 2.18 | 4 | .70 |
|  | HomACE | 13 | 26206.65 | 7694 | 10818.65 | 189.20 | 9 | 6.05E-36 |
| PHYSICAL FUNCTIONING | Sat | 25 | 23557.57 | 7682 | 8193.57 | - | - | - |
|  | Scalar Qual A | 24 | 23567.79 | 7683 | 8201.79 | - | - | - |
|  | Scalar Qual C | 24 | 23566.40 | 7683 | 8200.40 | - | - | - |
|  | **Scalar HetACE** | **20** | **23573.61** | **7687** | **8199.61** | 5.81 | 4 | .21 |
|  |  |  |  |  |  | 7.21 | 4 | .13 |
|  | HomACE | 14 | 23723.84 | 7693 | 8337.84 | 150.23 | 6 | 6.91E-30 |
| ROLE OF PHYSICAL  PROBLEMS | Sat | 25 | 32231.36 | 7682 | 16867.36 | - | - | - |
|  | Qual A | 26 | 32235.04 | 7681 | 16873.04 | - | - | - |
|  | Qual C | 26 | 32233.94 | 7681 | 16871.94 | - | - | - |
|  | **HetACE** | **22** | **32235.05** | **7685** | **16865.05** | <.001 | 4 | .99 |
|  |  |  |  |  |  | 1.11 | 4 | .89 |
|  | HomACE | 13 | 32414.89 | 7694 | 17026.89 | 179.84 | 9 | 5.47E-34 |
| EMOTIONAL WELLBEING | Sat | 25 | 24898.46 | 7682 | 9534.46 | - | - | - |
|  | Scalar  Qual A | 24 | 24901.51 | 7683 | 9535.51 | - | - | - |
|  | Scalar  Qual C | 24 | 24900.35 | 7683 | 9534.35 | - | - | - |
|  | **Scalar**  **HetACE** | **20** | **24905.34** | **7687** | **9531.34** | 3.83 | 4 | .43 |
|  |  |  |  |  |  | 4.99 | 4 | .29 |
|  | HomACE | 14 | 25055.10 | 7693 | 9669.10 | 149.76 | 6 | 8.71E-30 |
| ROLE OF EMOTIONAL PROBLEMS | Sat | 25 | 25305.41 | 7682 | 9941.41 | - | - | - |
|  | Qual A | 26 | 25308.27 | 7681 | 9946.27 | - | - | - |
|  | Qual C | 26 | 25307.90 | 7681 | 9945.90 | - | - | - |
|  | **HetACE** | **22** | **25309.09** | **7685** | **9939.09** | .83 | 4 | .93 |
|  |  |  |  |  |  | 1.20 | 4 | .88 |
|  | HomACE | 13 | 25478.03 | 7694 | 10090.03 | 169.77 | 9 | 1.03E-31 |
| ENERGY/  FATIGUE | Sat | 25 | 22887.11 | 7682 | 7523.11 | - | - | - |
|  | Scalar Qual A | 20 | 22896.19 | 7687 | 7522.19 | - | - | - |
|  | Scalar Qual C | 20 | 22893.92 | 7687 | 7519.92 | - | - | - |
|  | **HetACE** | **16** | **22899.55** | **7691** | **7518.55** | 4.36 | 4 | .36 |
|  |  |  |  |  |  | 6.62 | 4 | .16 |
|  | HomACE | 13 | 23043.55 | 7694 | 7655.55 | 144.98 | 6 | 8.50E-31 |
| PAIN | Sat | 25 | 29520.08 | 7683 | 14154.08 | - | - | - |
|  | Scalar  Qual A | 24 | 29533.35 | 7684 | 14165.35 | - | - | - |
|  | Scalar  Qual C | 24 | 29527.35 | 7684 | 14159.35 | - | - | - |
|  | **Scalar**  **HetACE** | **20** | **29536.74** | **7688** | **14160.74** | 3.39 | 4 | .50 |
|  |  |  |  |  |  | 9.39 | 4 | .05 |
|  | HomACE | 14 | 29687.87 | 7694 | 14299.87 | 151.13 | 6 | 4.45E-30 |
| SOCIAL FUNCTIONING | Sat | 25 | 16068.17 | 7723 | 622.17 | - | - | - |
|  | Qual A | 28 | 16073.10 | 7722 | 629.10 | - | - | - |
|  | Qual C | 28 | 16070.54 | 7722 | 626.54 | - | - | - |
|  | **HetACE** | **24** | **16077.83** | **7726** | **625.83** | 4.73 | 4 | .32 |
|  |  |  |  |  |  | 7.29 | 4 | .12 |
|  | HomACE | 15 | 16223.18 | 7735 | 753.18 | 145.35 | 9 | 8.11E-27 |

*Sat = Saturated phenotypic model; Qual A = Model testing for quantitative and qualitative sex differences in genetic influences ; Qual C = Model testing quantitative and qualitative sex differences in shared environmental influences; HetACE= Heterogeneity ACE model testing for quantitative sex differences only (magnitude of genetic and environmental influences on and across variables differs across sex); HomACE= Homogeneity ACE model (genetic and environmental influences on and across variables equated across sex). Note that the HetACE model was compared to the qualitative models (in the order of Qual A then Qual C respectively) and the homogeneity model was compared to the HetACE model. Also note that for variables physical functioning, emotional wellbeing, pain and energy/fatigue a hybrid scalar bivariate model was fit, to allow for variance differences (but no standardized ACE differences) in these variables and aetiological sex differences for anxiety. Ep= estimated parameters; -2LL= -2 Log Likelihood; df= degrees of freedom; AIC = Akaike’s Information Criterion (lower values indicate a better fit) ; ΔLL= Difference in -2log likelihood; Δdf= Difference in degrees of freedom; p= p-value. Best-fitting models are indicated in bold.*

**Supplementary table VII.** C correlations obtained from the Qualitative C model for the anxiety – pain analyses indicating marginal qualitative sex differences in C (with 95% CIs)

|  | PAIN |
| --- | --- |
| Rco11  C1m – C1f | .20  (-.61 / .86) |
| Rco21  C1m – C2f | -.18  (-.99 / 1) |
| Rco12  C2m – C1f | .11  (-.50 / .78) |
| Rco22  C2m – C2f | .16  (-1 / 1) |

*Rco11 = male-female correlation between the C factors of the SF-36 variable, C1m-C1f. Rco21 = correlation between the C factors of the SF-36 variable in males and the anxiety in females , C1m-C2f. Rco12 = correlation between the C factors of anxiety in males and SF-36 variable in females , C2m-C1f. Rco22 = male-female correlation between the C factors of anxiety, C2m-C2f. These correlations are obtained from the Qualitative C model which indicated sex differences in C. Correlations are non-significant, indicated by the 95% confidence intervals crossing zero.*
